# Direct wavefront sensing for high-resolution *in vivo* imaging in scattering tissue

**DOI:** 10.1038/ncomms8276

**Published:** 2015-06-15

**Authors:** Kai Wang, Wenzhi Sun, Christopher T. Richie, Brandon K. Harvey, Eric Betzig, Na Ji

**Affiliations:** 1 Janelia Research Campus, Howard Hughes Medical Institute, 19700 Helix Drive, Ashburn, Virginia 20147, USA; 2 Intramural Research Program, National Institute on Drug Abuse, 251 Bayview Boulevard, Baltimore, Maryland 21224, USA

## Abstract

Adaptive optics by direct imaging of the wavefront distortions of a laser-induced guide star has long been used in astronomy, and more recently in microscopy to compensate for aberrations in transparent specimens. Here we extend this approach to tissues that strongly scatter visible light by exploiting the reduced scattering of near-infrared guide stars. The method enables *in vivo* two-photon morphological and functional imaging down to 700 μm inside the mouse brain.

## Abstract (web summary)

Direct wavefront sensing with laser ‘guide stars' is used in astronomy and microscopy to correct for optical aberrations. Wang *et al.* use near-infrared guide stars to extend this approach to the highly scattering mouse brain, allowing high-resolution fluorescence imaging at 700μm depth.

## Graphical abstract

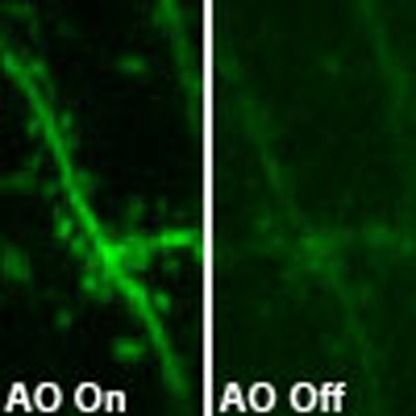

Optical microscopy, the primary approach used to obtain subcellular information noninvasively inside living specimens, is limited in depth and resolution by scattering and aberration in tissue such as the mammalian brain. For example, for *in vivo* two-photon excitation (TPE) fluorescence microscopy, the intensity of the focus formed by the ballistic component of the excitation light decreases exponentially with imaging depth^1^. Aberrations further prevent the surviving ballistic photons from forming a diffraction-limited focus, degrading signal, imaging contrast and resolution^2^. Longer wavelength excitation alleviates the scattering problem^3^ but the aberrations remain. To restore optimal imaging performance at depth, adaptive optical (AO) correction of aberrations in scattering tissue is essential^4^,^5^,^6^,^7^,^8^.

Recently, we demonstrated a simple, fast and photon-efficient method of aberration correction that recovers diffraction-limited performance in optically transparent samples such as the zebrafish embryo^9^. A direct-wavefront-sensing approach, it uses a Shack–Hartman (SH) sensor to measure the wavefront distortion of a fluorescent guide star (GS)^10^ created inside the specimen by TPE^11^. In the SH sensor, a lenslet array creates an array of images of the GS on a camera from different beamlets emerging from the back aperture of a microscope objective. Deviations in the positions of these images from the ideal array reflect aberrations experienced by the fluorescence light and are used to measure the wavefront. This information is then used by a wavefront modulating element to compensate for the aberrations in either TPE or single-photon confocal imaging modes.

For this method to work, a clear image of the GS is needed in each element of the sensor. However, light scattering in samples such as the mammalian brain not only reduces the ballistic fluorescence rays that form the GS images but also generates a diffuse background, which eventually overwhelms the ballistic component and makes it impossible to measure the aberration accurately. Scattering, however, is strongly dependent on the wavelength of the light and is much reduced at longer wavelengths. Here we demonstrate that, by employing a near-infrared (NIR) GS, direct wavefront sensing can be extended down to 700-μm depth in the mouse cortex *in vivo*.

## Results

### Direct wavefront sensing for morphological imaging at depth

**Figure 1:**
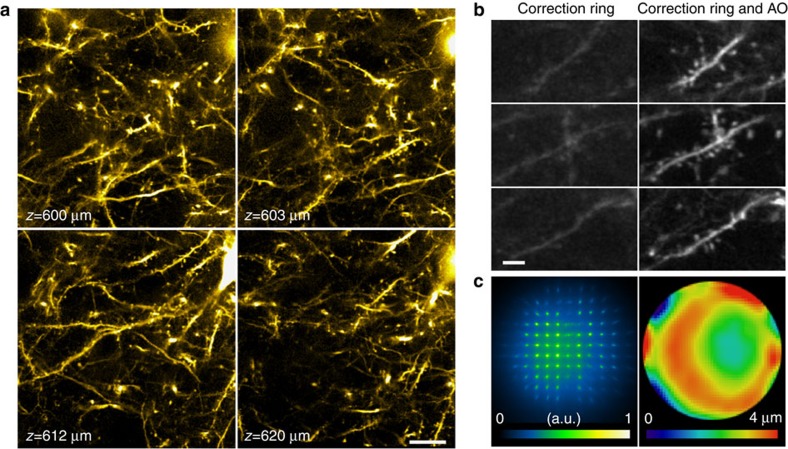
AO correction via direct wavefront sensing improves morphological imaging deep inside the cortex of a living mouse. (**a**) 120 × 120 μm field-of-view single-plane TPE fluorescence images of neurons in a Thy1-YFPH mouse at 600–620 μm below pia after AO correction. Scale bar, 20 μm. (**b**) TPE fluorescence images of dendrites at 606, 606.5 and 608.5-μm depth taken with objective correction ring adjustment only (left) and correction ring adjustment plus AO (right). Scale bar, 5 μm. (**c**) SH sensor image (left) for an NIR GS produced by TPE excitation of directly injected ICG, and the corresponding corrective wavefront (right). Representative images from >500 image sections in five mice.

Reduced scattering at longer wavelength allows direct wavefront sensing at increasingly greater depths in the scattering mouse brain *in vivo*, as demonstrated by GSs with emission peaks of 530 (YFP), 580 (tdTomato) and 810 nm (indocyanine green, ICG; Supplementary Figs 1 and 2; Methods). In one example, we injected ICG in the mouse cortex and used TPE to generate a NIR GS for aberration-corrected *in vivo* morphological imaging of layer 5 pyramidal neurons sparsely labelled with YFP (Methods). For *in vivo* mouse brain imaging, cranial window introduces aberrations that can be corrected either by AO (Supplementary Fig. 3) or by adjusting the correction ring on the objective (Supplementary Fig. 4). We used the correction ring of a 1.1 numerical aperture (NA) water-immersion objective to pre-correct cranial-window-induced aberrations (Supplementary Fig. 4), which ensured that the subsequent AO correction predominantly reflected the aberration of the brain tissue itself. With a single wavefront measurement 600 μm below pia, dendritic spines could be clearly resolved over a large field of view (120 × 120 μm; Fig. 1a). Without the additional signal (up to 6 × ) afforded by AO, most of these spines were invisible (Fig. 1b). Resolution enhancement by AO was also obvious: the full width at half maximum of a dendritic spine neck 550 μm below pia was reduced from ∼660 nm before AO to ∼430 nm after AO (Supplementary Fig. 5). The corrective wavefront contained of large elements of coma, astigmatism and spherical aberration caused by the refractive index mismatch between brain tissue and water, as well as high-order modes resulting from the heterogeneity of the brain (Fig. 1c; Supplementary Fig. 6). This heterogeneity is also evident in the SH image, where individual GS images have complex intensity and shape variations (Fig. 1c). Our direct-wavefront-sensing approach can be applied to improve structural imaging at 700 μm (Supplementary Movie 1) or even 760 μm (Supplementary Movie 2) below pia, despite the presence of substantial brain motion at these depths. Even deeper corrections are limited by the cranial window size (Supplementary Fig. 7).

**Figure 2:**
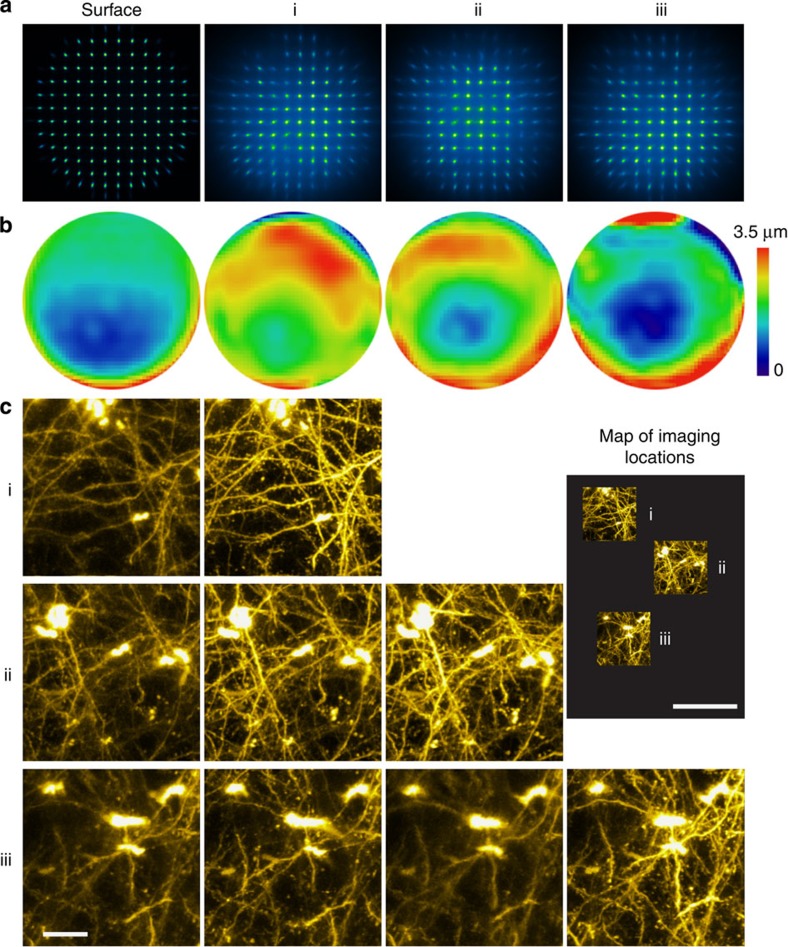
Exemplary spatial variation of sample-induced aberrations in the cortex of a living mouse. (**a**) SH images and (**b**) their corresponding corrective wavefronts obtained at pia surface and three different regions (i, ii and iii) 400 μm below pia of a Thy1-YFPH mouse. (**c**) Images of regions i, ii and iii obtained with the corrective wavefronts in **b**, respectively. Scale bar, 20 μm. The inset (‘map of imaging locations') shows the relative positions of regions i, ii and iii. Scale bar, 100 μm (inset).

Even though a single correction can improve image quality over a field of hundreds of microns in the mouse cortex^5^,^12^, the best performance is still obtained when the aberration is measured at the imaging location. To demonstrate, we measured aberrations at the surface of the mouse cortex as well as three locations at 400-μm depth that were laterally separated by 200–400 μm (Fig. 2). Although the three wavefronts measured at 400-μm depth were similar, in all three cases the highest signal and greatest contrast recovery occurred when the locally measured correction was employed. Therefore, to have diffraction-limited images over a large field of view (for example, 1 × 1 mm^2^), frequent AO corrections are likely needed. Our method is well suited for this eventuality, thanks to its high correction speed (milliseconds to seconds; Supplementary Table 1).

### Direct wavefront sensing for functional imaging at depth

**Figure 3:**
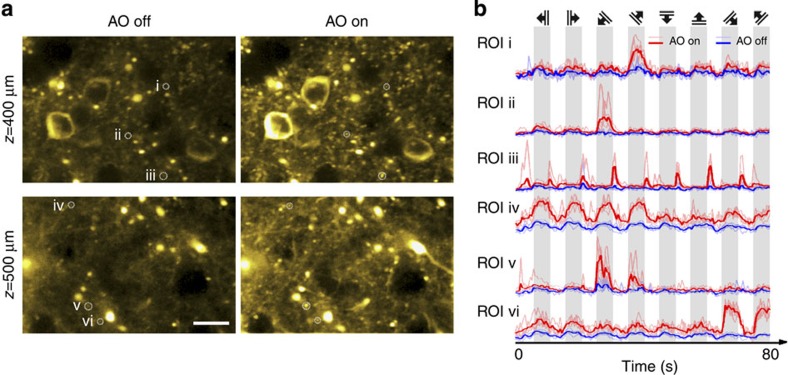
AO correction via direct wavefront sensing improves functional calcium imaging deep inside the cortex of a living mouse. (**a**) Calcium responses evoked by drifting-grating stimulation 400 and 500 μm below pia in the primary visual cortex of a mouse (Thy1-GCaMP6s line GP4.3) before (left panel) and after (right panel) correction. Brightness of each pixel reflects its s.d. across 800 frames imaged during five repetitions of the drifting-grating stimulus set, and is correlated with the local calcium transient magnitude. Scale bar, 20 μm. (**b**) Calcium transients at regions of interest (ROI) i–vi, before and after AO correction. Solid colours label averaged transients; faded colours label transients during specific repetitions. Top panel indicates the orientations and drifting directions of the grating stimuli. Representative images from >20 imaging sections in three mice.

Widely used in medical diagnostics^13^, injection of ICG allows this direct wavefront-sensing approach to be applied to a wide variety of tissues *in vivo*. In the mouse brain, it can improve not only morphological imaging but also the sensitivity when measuring neural activity at 500-μm depth in cells expressing the calcium indicator GCaMP6s^14^,^15^ (Thy1-GCaMP6s GP4.3 mouse; Supplementary Movie 3). One caveat, however, is that we observed heightened spontaneous activity of GCaMP6s in cortical regions injected with ICG. To eliminate this, we replaced ICG with the NIR protein iRFP NIR fluorescence protein iRFP713^16^ (peak emission at 713 nm) expressed via a viral vector in a subset of neurons in the primary visual cortex of the Thy1-GCaMP6s GP4.3 mice. With this marker, we were able to use a TPE-generated NIR GS to obtain corrective wavefronts down to 500 μm below pia, which then led to markedly improved sensitivity to evoked calcium responses in the visual cortex. Indeed, after AO correction, calcium transients could be detected in many more neurites at depths of 400 (Fig. 3; Supplementary Movie 4), 500 (Fig. 3; Supplementary Movie 5) and 600 μm (Supplementary Movie 6). Several factors contribute to these drastic improvements: calcium indicators usually have a heterogeneous labelling density in the brain. The more strongly labelled somata or neurites constitute the structures of interest, which are surrounded by the axons and dendrites of other more weakly labelled neurons. Calcium transients from these weakly labelled neuropil, or the ‘neuropil contaminations'^17^, represent the average response of many neurons and are not selective to the orientation of our visual stimuli. For a fine neurite, calcium transients within the laser focus are composed of signals from both the neurite and its surrounding neuropil. In a focus enlarged and dimmed by aberration, the calcium transient of the neurite may be overwhelmed by that of the neuropil. AO correction, however, both reduces the focal volume and increases the focal intensity, which enhances the neurite signal a lot more than the neuropil background. As a result, the orientation selectivity of many neurites is only detectable after AO correction raises the neurite calcium transients above that of the neuropil background^5^. This suggests that, to have an accurate characterization of neuronal responses at synaptic resolution, correction of brain-induced aberrations is essential.

### Direct wavefront sensing for confocal imaging in mouse brain

**Figure 4:**
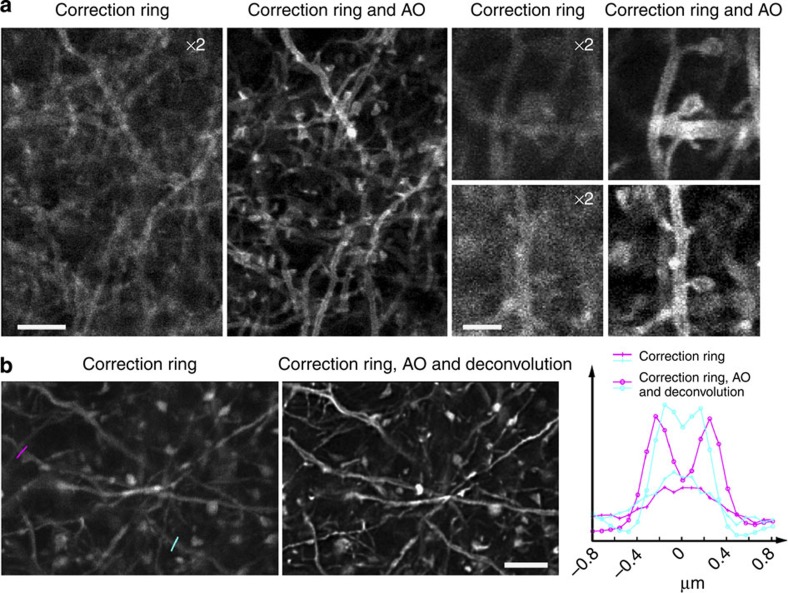
AO confocal imaging in the cortex of a living mouse by direct wavefront sensing of a GS generated by TPE excitation of neurons expressing YFP. (**a**) Single-plane confocal images of membrane-labelled neuronal processes ∼10 μm below pia of a mouse (Thy1-ChR2-EYFP) measured with objective correction ring only (left) and correction ring adjustment plus AO (right). The images without AO have been digitally enhanced 2 × in brightness to improve visibility. Scale bars, 5 μm (first column); 2 μm (third column). (**b**) Confocal images of neuronal processes 3–17 μm below pia having cytosolic expression of YFP in a Thy1-YFPH mouse measured with correction ring adjustment, but either no AO (left) or AO plus deconvolution (right). Line cuts at far right compare the signal strength and resolution in the two cases when imaging two voids (coloured lines at left) likely caused by displacement of cytosolic YFP by the mitochondria within the dendrites. Representative images from >100 imaging sections in two mice. Scale bar, 5 μm.

Although two-photon imaging is necessary to image fluorescence multiple scattering lengths deep within tissue, single-photon modalities such as confocal microscopy have intrinsically higher spatial resolution and can be applied in the superficial layers of the brain, where scattering is still low. Even here, however, AO can play an important role, as the multiple protective layers over the brain (for example, dura mater, arachnoid and pia mater) as well as slight tilt of the cranial window can impose substantial aberrations on both the excitation and emission light. Instead of painstakingly and invasively removing some of these protective layers^18^, our method can provide a quick AO correction using fluorescence from the same structures that are the target of the imaging. For example, we were able to use TPE to generate a descanned GS from membrane-specific EYFP expressed in apical dendrites in layer I of the mouse cortex (Thy1-ChR2-EYFP-transgenic mice). After AO correction, the dendritic branches and spines were clearly resolved as hollow, thanks to the membrane marker (Fig. 4a). With a different transgenic line (Thy1-YFPH) expressing cytosolic YFP in a subset of neurons, dark voids were occasionally observed in apical dendrites, possibly caused by mitochondria displacing the cytosolic YFP (Fig. 4b; Supplementary Movie 7). Thus, even in these superficial layers, AO can substantially improve imaging quality *in vivo*.

## Discussion

Even though wavefront measurement can be achieved with a stationary GS, in our experiments, the NIR GS was scanned over a small area (<60 × 60 μm^2^; Supplementary Tables 1–4), which reduces photobleaching and gives rise to a higher quality spot pattern on the wavefront sensor^9^. In principle, as long as the area used for wavefront measurement is smaller than the isoplanatic patch of the tissue, scanning the GS does not compromise AO performance. In practice, the optimal scanning field of view used for wavefront measurement depends on properties of the fluorophore (for example, its photophysical and photochemical properties as well as labelling density), the tissue and the nature of the experiment, thus, should be determined heuristically.

With an NIR GS, our approach is not only less sensitive to scattering but also to absorption by haemoglobin (Supplementary Fig. 8), which otherwise poses a major challenge for a visible (VIS) GS. With typical expression levels of common fluorescent labels and sensors, the method obtains a correction in the range of <100 ms to several seconds, and is insensitive to sample movement or variations in fluorescence intensity (for example, photobleaching, calcium or voltage transients). Immediately applicable to medical imaging preparations where NIR dyes are routinely introduced for contrast, this method will benefit further with the continued improvement of red and NIR fluorescent proteins as morphological and functional probes^19^,^20^,^21^.

## Methods

### Scanning AO microscope with direct wavefront sensing

The system (Supplementary Fig. 9) has three working modes: AO correction, TPE imaging and confocal imaging. In the AO correction and TPE imaging modes (Supplementary Fig. 9a), NIR excitation from a pulsed laser (Coherent, Chameleon Ultra II or Spectra-Physics, InSight DeepSee) is expanded and collimated by a pair of lens (*F*_1_=50 mm and *F*_2_=500 mm). It is coupled into the system by a dichroic mirror D3 (Semrock, FF670-SDi01-25x36) placed between these two lenses. The collimated NIR beam slightly overfills the aperture of the deformable mirror (DM, Alpao, DM 97-15) and is reflected with shaped wavefront. The DM is conjugated to two scanning galvo mirrors Galvo X and Y (Cambridge Technology, 6215H) and the back pupil of the objective (Nikon, CFI Apo LWD 25XW, 1.1 NA and 2 mm working distance (WD)) sequentially using three pairs of achromatic relay lenses operating in the 2*F*_1_+2*F*_2_ configuration (focal lengths: *F*_1_=300 mm and *F*_2_=100 mm between DM and Galvo Y; *F*_1_=85 mm and *F*_2_=85 mm between Galvo Y and X; and *F*_1_=100 mm and *F*_2_=400 mm between Galvo X and the objective rear pupil). The objective focuses the TPE beam into the sample and collects the excited fluorescence. For TPE imaging, the fluorescence is reflected by a dichroic mirror D1 (Semrock, FF665-Di02-25x36) placed immediately after the objective and focused by a lens (focal length: *f*=75 mm) onto a photomultiplier tube PMT1 (Hamamatsu, H7422-40). In the AO correction mode, the fluorescence from the TPE GS is descanned by Galvos X and Y and separated from the excitation light by dichroic mirror D2 (Semrock, FF875-Di01-25x36), before being relayed by a pair of lenses (*F*_1_=100 mm and *F*_1_=150 mm) to a lenslet array (Edmund Optics, 64–483) conjugate with the objective rear pupil. This array serves as the first part of the SH sensor. The SH camera (Andor iXon3 897 EMCCD) is placed at the focal plane of the lenslet array to record an array of images of the GS. In two-photon imaging mode, the AO system is operated in an open-loop manner where the DM only controls the wavefront of the excitation light but is not in the light path of the GS fluorescence. In the confocal imaging mode (Supplementary Fig. 9b), the VIS excitation is introduced into system by a dichroic mirror D4 (Semrock, Di01-R488/561-25x36) and follows the same light path as NIR excitation in the AO and TPE mode. The dichroic mirrors D1 and D2 are changed to D1′ and D2′ (Semrock, FF409-Di03-25x36) to allow VIS light transmission. The emitted fluorescence follows the reverse path of the excitation, passes through the dichroic mirror D4 and is collected by photomultiplier tube PMT2. A 30-μm diameter pinhole (Thorlabs, P30S) located at the focus of the fluorescence is placed in front of the PMT2 for confocal imaging. Various band-pass optical filters F1, F2 and F3 are placed in front of PMT1, the SH sensor and PMT2 according to the wavelengths of the working fluorophore.

### Calibration of the DM

The DM is calibrated separately before being integrated into the system. The actuator influence functions of the DM are measured using a home-built Michelson interferometer. Each influence function is expanded into Zernike polynomials. On the basis of the measured influence functions, the actuators' voltage settings for the first 55 Zernike modes can be obtained using a generalized matrix inversion method. In this way, the shape of the DM can be controlled deterministically through linear combinations of these modes.

### Calibration of the SH sensor

The SH sensor is calibrated with the DM in the system to minimize errors caused by misalignment. During the calibration, the objective is removed and a flat mirror is placed at the back pupil plane of the objective, which is conjugated to both the DM and SH sensor. An attenuated NIR beam is launched into the optical system and reflected by the DM with its wavefront shaped. Then, it is back reflected by the inserted mirror and directed to the SH sensor, where the wavefront changes introduced by the DM can be detected by the SH sensor. In total, 55 SH measurements are taken while applying the first 55 Zernike modes to the DM. Spot shifts determined in the 55 SH measurements form the basis with which SH measurements from *in vivo* samples can be decomposed. Due to the descanning and averaging over a volume, aberrations measured in these samples are mostly composed of low-order Zernike modes. Therefore, the modal phase reconstruction strategy is adopted to make wavefront sensing more robust against noise and missing spots in the SH measurements.

### System characterization

The principles of system aberration correction, wavefront sensing, image acquisition, control electronics and control software are the same as those used in our earlier work^9^ except that the two spatial light modulators working for VIS and NIR beams separately are now replaced by a single DM, which not only simplifies the system but also works over a wider wavelength range. All experiments started after compensation for system aberration, therefore, all of the wavefronts presented here represent the corrections for sample-induced aberrations.

### Comparison of GSs of different wavelengths

We compared three different fluorophores in the cortex of living mice: YFP in a subset of layer 5 pyramidal neurons, tdTomato in a majority of cortical neurons and directly injected ICG (Supplementary Fig. 1). The emission peaks of these fluorophores are around 530, 580 and 810 nm, respectively. We measured the signal-to-background ratio (SBR) of individual GS images from the recorded SH sensor images (Supplementary Fig. 2a,b). Within a given SH image, SBR varies due to the inhomogeneity of the tissue, and generally decreases for light rays that are at higher NA and thus experience larger scattering loss. By analysing all spots in each SH image, we find that tdTomato outperforms YFP at all depths because of its smaller extinction coefficient (Supplementary Fig. 2c). Even though it has a longer emission wavelength, ICG only outperforms tdTomato beyond a depth of 400 μm. At shallower depths, the SBR of ICG is reduced by out-of-focus background fluorescence due to direct linear excitation of the ICG by the 1,020 nm wavelength of the TPE laser. Nevertheless, ICG provides greater uniformity in the SBR at all depths for all GS spots across the sensor (Supplementary Fig. 2b), which is essential for reliable wavefront measurement. Even though the SBR is unaffected by the GS brightness *B*, a brighter GS can compensate for the decrease of SBR at depth (Supplementary Fig. 2d), since the signal from the ballistic component increases linearly with *B*, whereas the noise from the background increases only as 
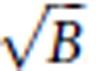
. Eventually, however, the brightness required for meaningful AO correction will become impractical to reach due to limitations imposed by sample damage, photobleaching, excessive scattering loss of marginal rays and unacceptably long measurement time. Beyond these limits, AO correction via indirect wavefront measurement^5^,^12^ is the only option.

### Animal care and preparation

All mouse experimental protocols were conducted according to the National Institutes of Health (NIH) guidelines for animal research and were approved by the Institutional Animal Care and Use Committee at Janelia Research Campus, Howard Hughes Medical Institute (names of mouse line for each experiment are stated in main text, figure legends and Supplementary Tables 1–4).

### Procedures for *in vivo* imaging

Mice 8 weeks or older were anaesthetized with isoflurane (1–2% by volume in O_2_) and given the analgesic buprenorphine (SC, 0.3 mg kg^−1^). Using aseptic technique, a 3.5-mm diameter craniotomy was made over the left cortex. Dura was left intact. For experiments with ICG as GS, 5 nl of ICG solution (0.1% in saline) was slowly injected into target imaging area with a glass pipette with a 15–20 μm opening bevelled at 45° and back filled with mineral oil. To introduce iRFP GS, 30 nl of AAV1-CaMKII-iRFP (Addgene #47903) virus solution (produced by the NIDA Optogenetics and Transgenic Technology Core facility) was injected into V1 (left hemisphere, 3.4 mm posterior to Bregma; 2.7 mm lateral from midline; 0.4 mm below pia). A titanium headpost was attached to the skull with cyanoacrylate glue and dental acrylic. We adopted the following practice for optimal imaging performance without AO: to minimize non-brain-induced aberration, a cranial window made of a single 170-μm-thick coverslip (Fisher Scientific no. 1) was embedded in the craniotomy and sealed in place with dental acrylic. (The final cranial window has a clear aperture of ∼3-mm diameter.) During imaging, the mouse head was carefully aligned to make the cranial window and the brain maximally perpendicular to the microscope objective optical axis, so that the spherical aberration caused by the cranial window can be largely compensated by the correction ring on the objective (Supplementary Fig. 4).

### *In vivo* functional imaging

When using an ICG GS, functional calcium imaging experiments immediately followed the injection, whereas a 3-week post-surgery recovery period was adopted for iRFP to reach sufficient expression level. Visual stimuli were presented to the eye contralateral to the hemisphere with craniotomy using a computer monitor. The stimuli were blue sinusoidal gratings at 100% contrast that drift at 2 Hz in eight different directions (0, 180, 45, 225, 90, 270, 135 and 315°) for 5 s (10 imaging frames) each. They were interleaved with 5 s of a black screen. For each imaging session, the sequence was repeated five times and a total 800 calcium imaging frames were collected.

## Additional information

**How to cite this article:** Wang, K. *et al.* Direct wavefront sensing for high-resolution *in vivo* imaging in scattering tissue. *Nat. Commun.* 6:7276 doi: 10.1038/ncomms8276 (2015).

## Supplementary Material

Supplementary Information Supplementary Figures 1-9, Supplementary Tables 1-4 and Supplementary References.

Supplementary Movie 1 *In vivo* two-photon images of neurons 700 μm below pia measured without and with AO correction. 3D image stacks of neurons expressing cytosolic YFP at 700 μm below pia. The corrective wavefront is measured at the same location as the imaging through TPE of directly injected ICG to produce an NIR guide star. Despite obvious motion artefacts, dendritic spines can be observed after AO correction.

Supplementary Movie 2 *In vivo* two-photon imaging of neurons 750 μm below pia measured without AO correction, and with AO correction at 700 μm. 3D image stacks of cytosolic YFP-labelled neurons at 750 μm below pia. The corrective wavefront is measured at 50 μm above the image location (i.e., correction in Supplementary Movie 1).

Supplementary Movie 3 *In vivo* two-photon functional imaging of spontaneous neuronal activity 500 μm below pia without and with AO correction. Two-photon time lapse images of GCaMP6s-expressing neurons 500 μm below pia of a transgenic mouse. The corrective wavefront is obtained at the image location.

Supplementary Movie 4 *In vivo* two-photon functional imaging of visually evoked neuronal activity 400 μm below pia without and with AO correction. Two photon time lapse images of GCaMP6s-expressing neurons 400 μm below pia in the visual cortex of a transgenic mouse that is presented with five repetitions of a drifting-grating stimulus set. The corrective wavefront is measured at the image location.

Supplementary Movie 5 *In vivo* two-photon functional imaging of visually evoked neuronal activitiy 500 μm below pia without and with AO correction. Two photon time lapse images of GCaMP6s-expressing neurons 500 μm below pia in the visual cortex of a transgenic mouse that is presented with five repetitions of a drifting-grating stimulus set. The corrective wavefront is measured at the image location.

Supplementary Movie 6 *In vivo* two-photon functional imaging of neuronal activity 600 μm below pia without and with AO correction. Two photon time lapse images of GCaMP6s-expressing neurons 600 μm below pia in the visual cortex of a transgenic mouse that is presented with five repetitions of a drifting-grating stimulus set. The corrective wavefront is measured 100 μm above the image location (i.e., correction in Supplementary Movie 5).

Supplementary Movie 7 *In vivo* confocal imaging of dendrites in layer I of the mouse cortex with AO correction. 3D image stack of neurons 0-86 μm below pia expressing cytosolic YFP, measured with the single photon confocal mode in vivo. The corrective wavefronts are measured by two photon excitation of YFP every 15 μm in z.
