# Supplementary material for: Direct wavefront sensing for high-resolution in vivo imaging in scattering tissue
Source: Nat Commun. 2015 Jun 15;6:7276. doi: 10.1038/ncomms8276 (PMC4490402; doi:10.1038/ncomms8276)
Supplement: Supplementary Information — Supplementary Figures 1-9, Supplementary Tables 1-4 and Supplementary References. [file ncomms8276-s1.pdf]

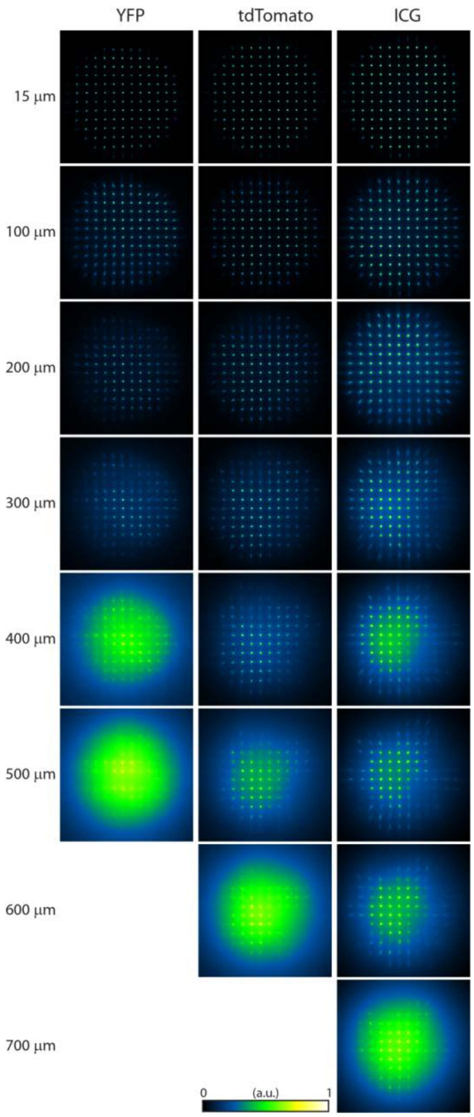

Supplementary Figure 1| Guide stars of progressively longer wavelengths can be used for direct wavefront sensing at increasingly large depth in the cortex of the living mouse. Typical SH images of guide stars obtained at different depths by TPE of: YFP in sparsely labeled L5 pyramidal neurons (left column); tdTomato in densely labeled cortical neurons (middle column); and the directly injected ICG (right column) in the cortex of a living mouse.

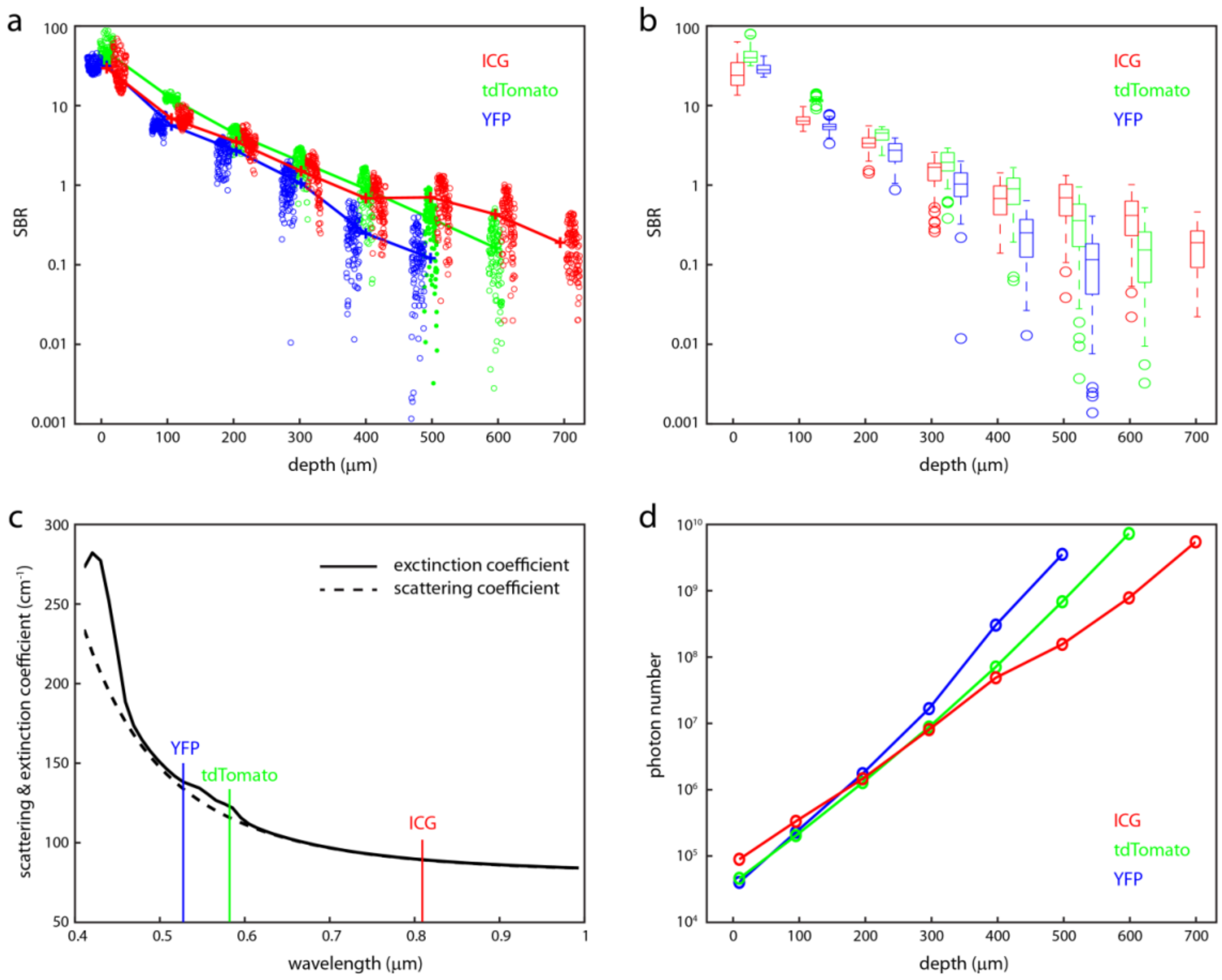

Supplementary Figure 2| Performance comparison of guide stars generated by TPE of YFP, tdTomato or IGC in the cortex of a living mouse. (a) Signal-to-background ratio (SBR) of guide-star images on the SH sensor (**Supplementary Figure 1**) generated by TPE of YFP, tdTomato, or ICG at different depths in the cortex of the living mouse. Each circle is the SBR of the guide-star image formed by a single lenslet. The averages of each data set at different depths are connected by solid lines. **(b)** Data in (a) represented as box-and-whisker plots (circles indicate outliers). **(c)** Scattering and extinction coefficients of the live mouse cortex at different wavelengths[1]. **(d)** The total number of fluorescence photons emitted within the cortex from the guide star that is required for an average spot localization precision of 1/5 pixel size on the SH sensor (1 pixel size equals 1.07 μm at sample plane). The calculation was done for a NA 1.1 objective using the extinction coefficients in (c). The localization precision is estimated based on the theory by Thompson, et al[2].

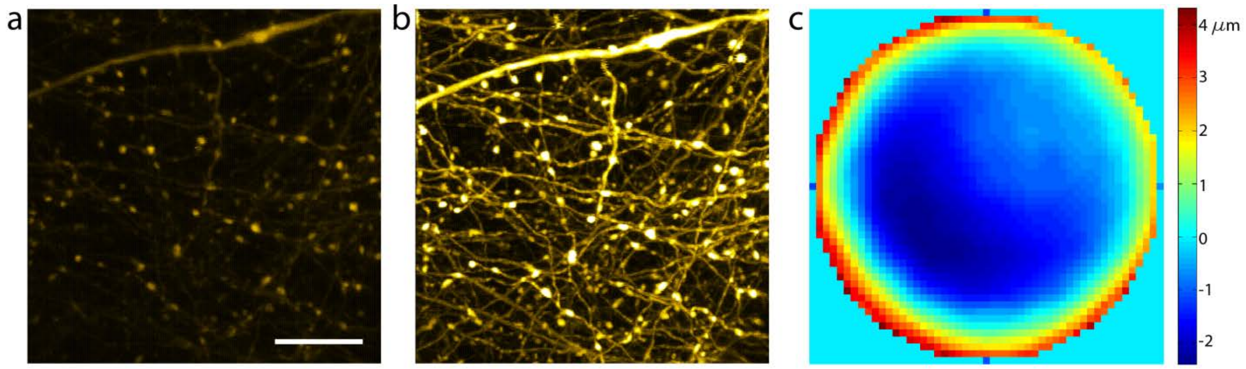

Supplementary Figure 3| Correction of cranial-window-induced aberration. (a-b) *In vivo* TPE fluorescence images of axons and dendrites at 15 μm depth in the cortex of a Thy1-YFPH mouse as acquired **(a)** without AO and **(b)** with AO corrections. The objective correction ring was adjusted to optimize imaging in water (i.e., without any correction for the cranial window aberration). Scale bar is 20 μm. **(c)** Corrective wavefront for the cranial-window-induced aberration.

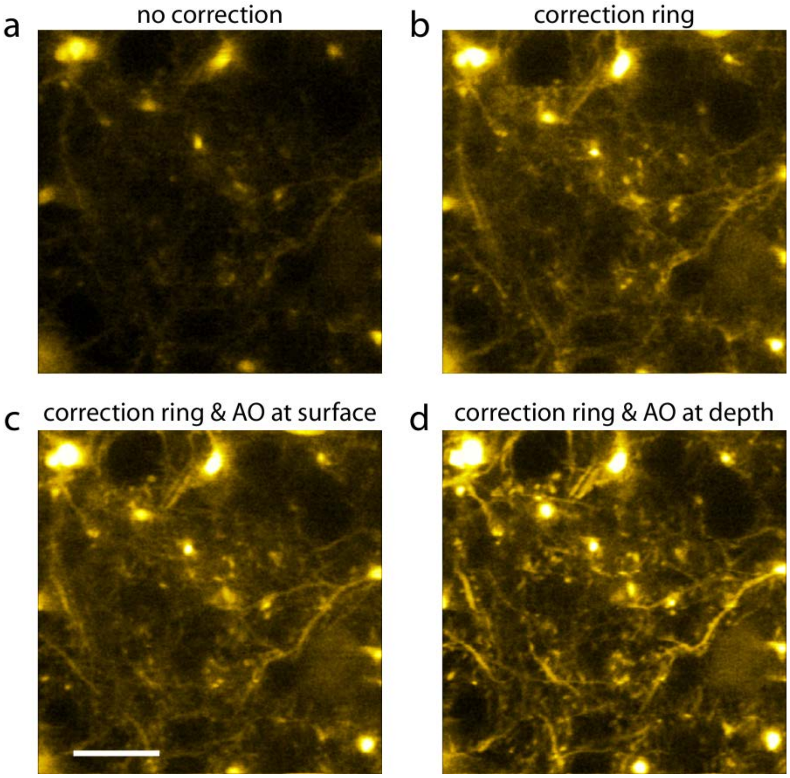

Supplementary Figure 4| Image comparison with different correction schemes. Comparative in vivo TPE fluorescence images of the same field of neurons 500 µm deep in the cortex of a Thy1-YFPH mouse as acquired with: **(a)** the objective correction ring optimized for imaging in water, and no AO used; **(b)** the objective correction ring optimized for imaging into water through a 170 µm thick cranial window, and no AO used; **(c)** same as (b), except with additional AO correction carried out on the surface of the cortex to correct any residual aberrations at that position; **(d)** same as (b), except with additional AO correction carried out at the 500 µm depth where the images were taken. Scale bar is 20 µm.

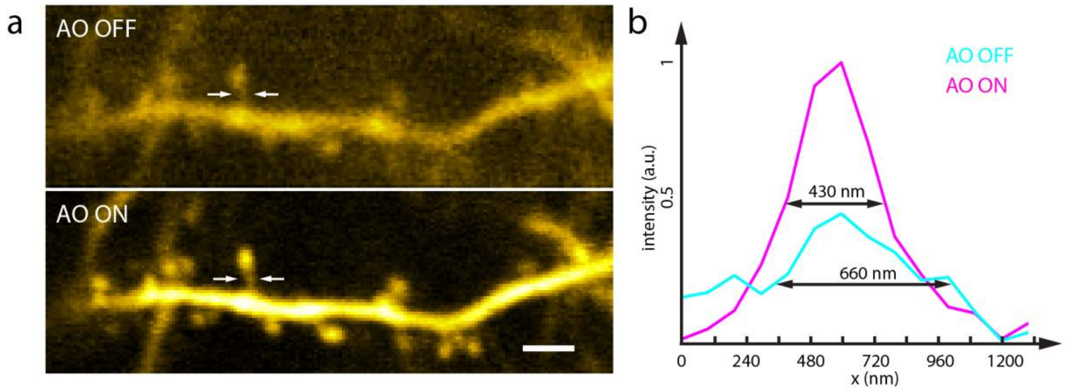

Supplementary Figure 5| Resolution enhancement after AO correction. (a) Single-plane TPE fluorescence images of a dendritic branch at 550 µm below pia in a Thy1-YFPH mouse before (with only correction ring) and after AO correction. Scale bar is 2 µm. **(b)** Line intensity profiles of the feature (the neck of a dendritic spine) indicated by arrows in (a). Image of the spine neck after AO correction has a full width at half maximum of 430 nm.

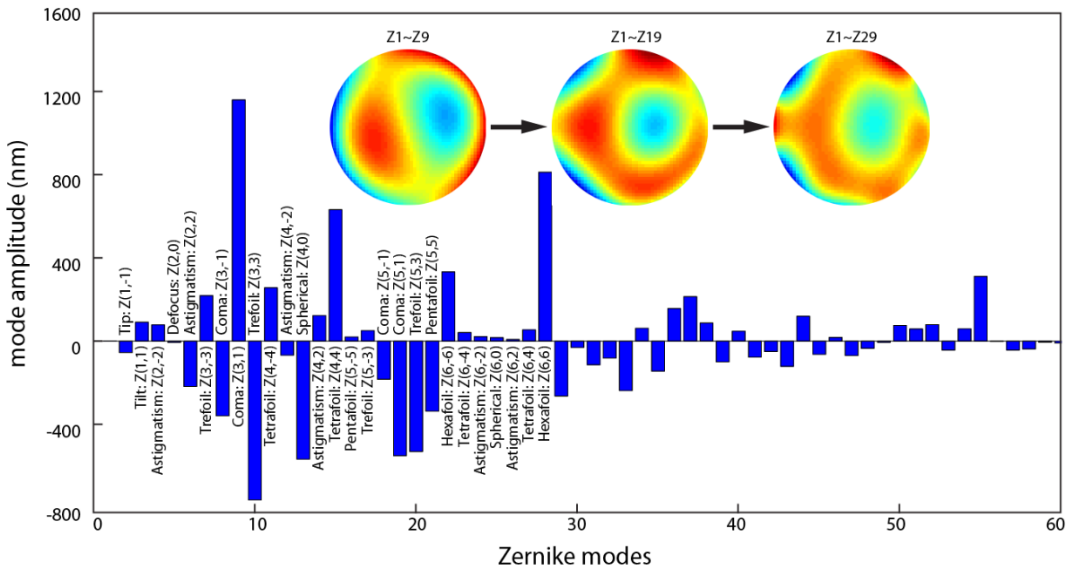

Supplementary Figure 6| Zernike modes for the corrective wavefront of Fig. 1c, measured at 600 µm depth in the mouse cortex. Amplitudes of Zernike modes in a modal decomposition of the wavefront. Inset shows the wavefront as calculated from the first 9, 19, or 29 modes, respectively.

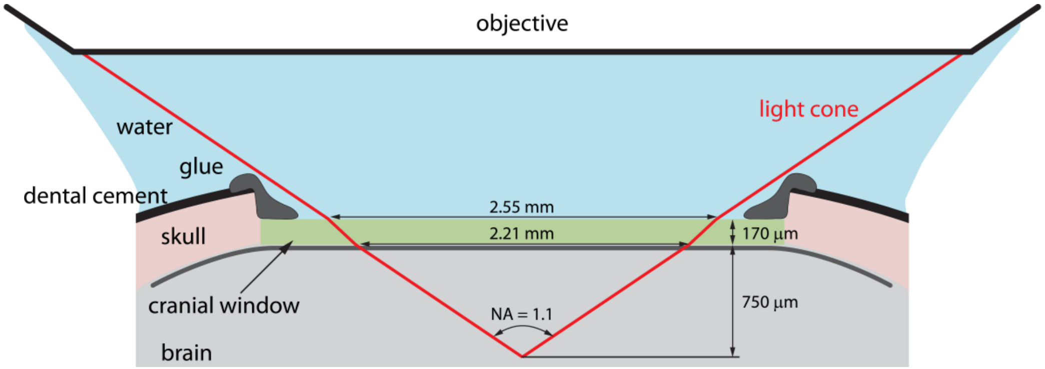

Supplementary Figure 7| Required cranial window diameter for imaging 750µm below pia with a 1.1 NA objective.

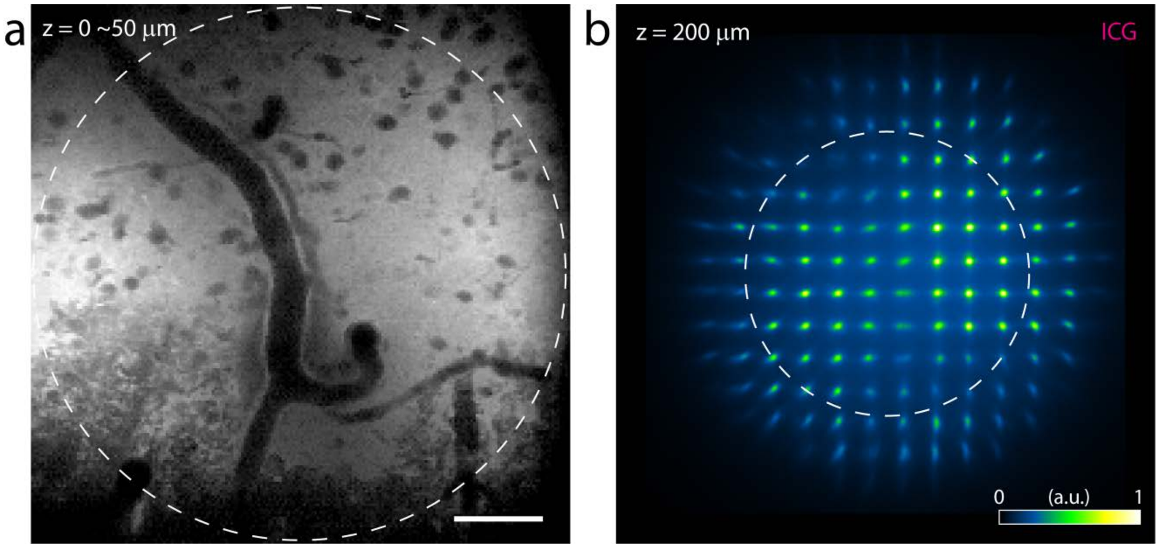

Supplementary Figure 8| Effects of hemoglobin absorption on direct wavefront sensing *in vivo*. (a) Blood (black) absorbs visible fluorescence, but **(b)** transmits NIR ICG fluorescence for wavefront sensing. The dashed circle in (a) corresponds to that in (b). Scale bar is 50 µm.

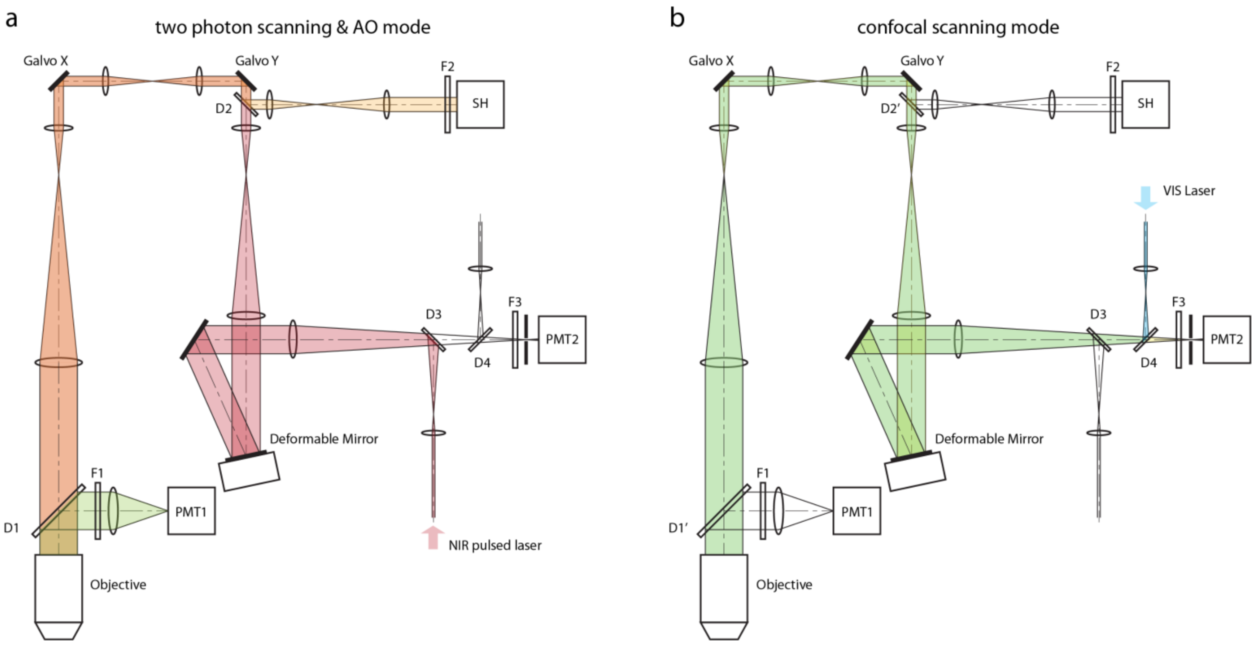

Supplementary Figure 9| Experimental schematics. (a) Two photon scanning & AO mode: Near-infrared (NIR) pulsed excitation (red) is directed into the system by dichroic mirror D3, reflected off a deformable mirror, and scanned by a pair of galvanometer mirrors (Galvo X and Y) before entering the rear pupil of a 1.1 NA water-dipping objective. The deformable mirror, Galvos X and Y, and the objective rear pupil are all mutually conjugate, so that the phase pattern from the deformable mirror is stationary at the rear pupil even while scanning. The emitted visible signal fluorescence is collected by the objective, reflected from dichroic mirror D1, and focused onto a photomultiplier tube PMT1. The NIR fluorescence from the TPE guide star (orange) is descanned by the galvos, separated from the NIR excitation by dichroic beamsplitter D2, and then sent to a Shack-Hartmann (SH) sensor to determine the aberrated wavefront of the emission. **(b)** Confocal scanning mode: visible excitation light (blue) is reflected by dichroic mirror D4 and follows the same light path as NIR excitation to the objective. The emission retraces the excitation path, except that it transmits through dichroic mirror D4 and enters another photomultiplier tube PMT2 after going through a pinhole that is conjugated to the objective focus.

Supplementary Table 1| Acquisition parameters for images in the main text.

Image property	Figure 1a and b	Figure 2	Figure 3a
Modality	two photon	two photon	two photon
Sample	B6.Cg-Tg(Thy1-YFPH)2Jrs/J	B6.Cg-Tg(Thy1-YFPH)2Jrs/J	C57BL/6J-Tg(Thy1-GCaMP6s)GP4.3Dkim/J
Fluorescent label	YFP (cytosolic)	YFP (cytosolic)	GCaMP6s (cytosolic)
Excitation wavelength (nm)	960	960	920
Excitation power post objective (mW)	56	16	60
Voxel volume (nm^3)/ Pixel size (nm^2)	200x200x500	200x200x500	250x250
Image volume (μm^3) / field (μm^2)	120x120x20	80x80x20	75x50
Pixel rate (pixels/s)	80k	80k	120k
Fluorophore for AO	ICG	Directly injected ICG	iRFP
AO correction excitation wavelength (nm)	1020	1020	920
AO correction excitation power (mW)	40	16	100
AO integration time (s)	2	2	2
AO correction field of view (μm^2)	60x60	60x60	60x60
additional comments	Gaussian blur with sigma of 1 (ImageJ) is applied	The image is the maximum intensity projection of imaging volume and Gaussian blur with sigma of 1 (ImageJ) is applied	none

Supplementary Table 1 (cont'd)| Acquisition parameters for images in the main text.

Image property	Figure 3b	Figure 4a	Figure 4b
Modality	two photon	confocal	confocal
Sample	C57BL/6J-Tg(Thy1-GCaMP6s)GP4.3Dkim/J	B6.Cg-Tg(Thy1-COP4/EYFP)18Gfng/J	B6.Cg-Tg(Thy1-YFPH)2Jrs/J
Fluorescent label	GCaMP6s (cytosolic)	YFP (membrane-expressed, fused to ChR2)	YFP (cytosolic)
Excitation wavelength (nm)	920	488	488
Excitation power (mW)	100	0.05	0.01
Voxel volume (nm^3)	250x250	80x80	80x80x270
Image volume (μm^3) / field (μm^2)	75x50	24x32	32x23
Pixel rate (pixels/s)	120k	80k	80k
Fluorophore for AO	iRFP	YFP	YFP
AO correction excitation wavelength (nm)	920	960	960
AO correction excitation power (mW)	120	2	2
AO integration time (s)	2	0.4	0.1
AO correction field of view (μm^2)	60x60	40x40	40x40
additional comments	none	none	Maximum intensity projection of three selected planes from the imaging volume. It's associated with Supplementary Movie 7

Supplementary Table 2| Acquisition parameters for supplementary figure 1.

**Image property**		**Figure 1 Left column**	**Figure 1 Middle column**	**Figure 1 Right column**
Sample		B6.Cg-Tg(Thy1-YFPH)2Jrs/J	NPY(65)-Cre X Ai9	Wild type
Fluorescent label		YFP (cytosolic)	tdTomato (cytosolic)	Directly injected ICG
AO correction excitation wavelength (nm)		960	1020	1020
AO integration time (s)		40	40	20
AO correction excitation power post objective (mW)	15 μm	6	3	1
	100 μm	22	7	12
	200 μm	52	12	27
	300 μm	86	27	45
	400 μm	117	45	62
	500 μm	133	62	76
	600 μm	-	87	87
	700 μm	-	-	87

Supplementary Table 3| Acquisition parameters for images in supplementary figures.

Image property	Figure 3	Figure 4	Figure 5
Modality	two photon	two photon	two photon
Sample	B6.Cg-Tg(Thy1-YFPH)2Jrs/J	B6.Cg-Tg(Thy1-YFPH)2Jrs/J	B6.Cg-Tg(Thy1-YFPH)2Jrs/J
Fluorescent label	YFP (cytosolic)	YFP (cytosolic)	YFP (cytosolic)
Excitation wavelength (nm)	960	960	960
Excitation power post objective (mW)	4	56	60
Voxel volume (nm^3)/ Pixel size (nm^2)	200x200x500	200x200	120x120
Image volume (μm^3) / field (μm^2)	80x80x15	80x80	24x12
Pixel rate (pixels/s)	80k	80k	120k
Fluorophore for AO	Directly injected ICG	Directly injected ICG	Directly injected ICG
AO correction excitation wavelength (nm)	960	1020	1020
AO correction excitation power (mW)	2	2 (surface)<br>40 (depth of 500 µm)	60
AO integration time (s)	1	0.2 (surface)<br>2 (depth of 500 µm)	2
AO correction field of view (μm^2)	40x40	60x60	60x60
additional comments	Maximum intensity projection of the imaging volume.	Gaussian blur with sigma of 1 (ImageJ)	The image is the average of 5 frames to increase SNR

Supplementary Table 4| Acquisition parameters for images in supplementary movies.

Image property	Movie 1	Movie 2	Movie 3
Modality	two photon	two photon	two photon
Sample	B6.Cg-Tg(Thy1-YFPH)2Jrs/J	B6.Cg-Tg(Thy1-YFPH)2Jrs/J	C57BL/6J-Tg(Thy1-GCaMP6s)GP4.3Dkim/J
Fluorescent label	YFP (cytosolic)	YFP (cytosolic)	GCaMP6s (cytosolic)
Excitation wavelength (nm)	960	960	920
Excitation power (mW)	56	87	90
Voxel volume (nm^3)/ Pixel size (nm^2)	200x200x500	200x200x500	400x400
Image volume (μm^3) / field (μm^2)	80x80x20	80x80x15	100x100
Pixel rate (pixels/s)	80k	40k	125k
Fluorophore for AO	Directly injected ICG	Directly injected ICG	Directly injected ICG
AO correction excitation wavelength (nm)	1020	N.A.	1020
AO correction excitation power (mW)	70	N.A.	40
AO integration time (s)	4	N.A.	10
AO correction field of view (μm^2)	60x60	N.A.	60x60
additional comments	none	none	none

Supplementary Table 4 (cont'd 1)| Acquisition parameters for images in supplementary movies.

Image property	Movie 4	Movie 5	Movie 6
Modality	two photon	two photon	two photon
Sample	C57BL/6J-Tg(Thy1-GCaMP6s)GP4.3Dkim/J	C57BL/6J-Tg(Thy1-GCaMP6s)GP4.3Dkim/J	C57BL/6J-Tg(Thy1-GCaMP6s)GP4.3Dkim/J
Fluorescent label	GCaMP6s (cytosolic)	GCaMP6s (cytosolic)	GCaMP6s (cytosolic)
Excitation wavelength (nm)	920	920	920
Excitation power (mW)	60	100	140
Voxel volume (nm^3)/ Pixel size (nm^2)	250x250	250x250	250x250
Image volume (μm^3) / field (μm^2)	75x50	75x50	75x50
Pixel rate (pixels/s)	120k	120k	120k
Fluorophore for AO	iRFP	iRFP	N.A.
AO correction excitation wavelength (nm)	920	920	N.A.
AO correction excitation power (mW)	100	120	N.A.
AO integration time (s)	2	2	N.A.
AO correction field of view (μm^2)	60x60	60x60	N.A.
additional comments	none	none	none

Supplementary Table 4 (cont'd 2)|Acquisition parameters for images in supplementary movies.

Image property	Movie 7
Modality	confocal
Sample	B6.Cg-Tg(Thy1-YFPH)2Jrs/J
Fluorescent label	YFP (cytosolic)
Excitation wavelength (nm)	488
Excitation power (mW)	from 0.01 to 0.1 with increasing depth
Voxel volume (nm^3)/ Pixel size (nm^2)	80x80x270
Image volume (μm^3) / field (μm^2)	32x32x86.4
Pixel rate (pixels/s)	80k
Fluorophore for AO	YFP
AO correction excitation wavelength (nm)	960
AO correction excitation power (mW)	2
AO integration time (s)	0.1
AO correction field of view (μm^2)	40x40
additional comments	Images at different depth are intensity-normalized

Supplementary References:

1. Jacques, S.L. Optical properties of biological tissues: a review. *Phys Med Biol* **58**, R37-61 (2013).
2. Thompson, R.E., Larson, D.R. & Webb, W.W. Precise nanometer localization analysis for individual fluorescent probes. *Biophys J* **82**, 2775-2783 (2002).
